# Correction

**Published:** 2008-06

Information was printed inaccurately in the 'How to’ article on applanation tonometry in Issue 64, December 2007. The corrections are shown here in **bold.**

## Preparation

- Ensure that the prism has been **disinfected** with isopropyl alcohol 70 per cent (methylated spirit) or sodium hypochlorite **1 per cent. The prism must be rinsed in sterile water** and wiped dry with a clean swab (as residue of the disinfectant may cause a caustic burn on the cornea).

## Method

When the applanation tonometry procedure has been completed:

- Wipe the prism with a clean, dry swab and replace it in the receptacle containing the **disinfectant.**

